# Cytosolic Peroxidases Protect the Lysosome of Bloodstream African Trypanosomes from Iron-Mediated Membrane Damage

**DOI:** 10.1371/journal.ppat.1004075

**Published:** 2014-04-10

**Authors:** Corinna Hiller, Amrei Nissen, Diego Benítez, Marcelo A. Comini, R. Luise Krauth-Siegel

**Affiliations:** 1 Biochemie-Zentrum der Universität Heidelberg (BZH), Heidelberg, Germany; 2 Group Redox Biology of Trypanosomes, Institut Pasteur de Montevideo, Montevideo, Uruguay; Washington University School of Medicine, United States of America

## Abstract

African trypanosomes express three virtually identical non-selenium glutathione peroxidase (Px)-type enzymes which preferably detoxify lipid-derived hydroperoxides. As shown previously, bloodstream *Trypanosoma brucei* lacking the mitochondrial Px III display only a weak and transient proliferation defect whereas parasites that lack the cytosolic Px I and Px II undergo extremely fast lipid peroxidation and cell lysis. The phenotype can completely be rescued by supplementing the medium with the α-tocopherol derivative Trolox. The mechanism underlying the rapid cell death remained however elusive. Here we show that the lysosome is the origin of the cellular injury. Feeding the *px I–II* knockout parasites with Alexa Fluor-conjugated dextran or LysoTracker in the presence of Trolox yielded a discrete lysosomal staining. Yet upon withdrawal of the antioxidant, the signal became progressively spread over the whole cell body and was completely lost, respectively. *T. brucei* acquire iron by endocytosis of host transferrin. Supplementing the medium with iron or transferrin induced, whereas the iron chelator deferoxamine and apo-transferrin attenuated lysis of the *px I–II* knockout cells. Immunofluorescence microscopy with MitoTracker and antibodies against the lysosomal marker protein p67 revealed that disintegration of the lysosome precedes mitochondrial damage. *In vivo* experiments confirmed the negligible role of the mitochondrial peroxidase: Mice infected with *px III* knockout cells displayed only a slightly delayed disease development compared to wild-type parasites. Our data demonstrate that in bloodstream African trypanosomes, the lysosome, not the mitochondrion, is the primary site of oxidative damage and cytosolic trypanothione/tryparedoxin-dependent peroxidases protect the lysosome from iron-induced membrane peroxidation. This process appears to be closely linked to the high endocytic rate and distinct iron acquisition mechanisms of the infective stage of *T. brucei*. The respective knockout of the cytosolic *px I–II* in the procyclic insect form resulted in cells that were fully viable in Trolox-free medium.

## Author Summary

In many cell types, mitochondria are the main source of intracellular reactive oxygen species but iron-induced oxidative lysosomal damage has been described as well. African trypanosomes are the causative agents of human sleeping sickness and the cattle disease Nagana. The parasites are obligate extracellular pathogens that multiply in the bloodstream and body fluids of their mammalian hosts and as procyclic forms in their insect vector, the tsetse fly. Bloodstream *Trypanosoma brucei* in which the genes for cytosolic lipid hydroperoxide-detoxifying peroxidases have been knocked out undergo an extremely rapid membrane peroxidation and lyse within less than two hours when they are cultured without an exogenous antioxidant. Here we show that the primary site of intracellular damage is the single terminal lysosome of the parasites. Disintegration of the lysosome clearly precedes damage of the mitochondrion and parasite death. Iron, acquired by the endocytosis of iron-loaded host transferrin, induces cell lysis. Contrary to the cytosolic enzymes, the respective mitochondrial peroxidase is dispensable for both *in vitro* proliferation and mouse infectivity. This is the first report demonstrating that cytosolic thiol peroxidases are responsible for protecting the lysosome of a cell.

## Introduction

In many tissues, the mitochondrial electron transport chain constitutes the primary source of endogenously produced superoxide anion, the precursor molecule of most reactive oxygen species [1], [2]. Hydrogen peroxide and lipid hydroperoxides formed as products are primarily removed by glutathione peroxidases (GPxs) [3]. Among the eight GPxs described in mammals, GPx4 is the only one that accepts phospholipid hydroperoxides as substrates even within intact biomembranes [4]. Another organelle that plays a critical role in oxidant-induced cell damage is the lysosome [5]. Intralysosomal iron, which probably represents the major fraction of cellular redox-active iron, can catalyze the peroxidation of membrane lipids. Once lysosomal rupture has occurred, the cell is irreversibly committed to death [6].

African trypanosomes, the causative agents of human sleeping sickness and Nagana cattle disease, are extracellular parasitic protozoa with a digenetic life cycle. *Trypanosoma brucei* multiply as infective bloodstream (BS) forms in the blood and body fluids of their mammalian hosts and as procyclic insect form in the midgut of the tsetse fly vector. Trypanosomes possess mitochondria and lysosomes as single copy organelles. The mitochondrion of the BS parasites is functionally repressed and the cells rely exclusively on glycolysis for ATP production [7]. Nevertheless, the organelle plays a crucial role by harbouring the alternative oxidase, the final acceptor of reducing equivalents generated during glycolysis, as well as the machinery for iron sulfur cluster biogenesis [8], [9]. BS *T. brucei* have one of the highest endocytic rates ever measured [10]. All vesicular trafficking of macromolecules into or out of the parasites takes place at the flagellar pocket which is the only area of the cell surface with endocytic activity. Both fluid-phase and receptor-mediated cargo enter the parasite via an early endosomal compartment, pass on to a recycling endosome, and are ultimately delivered to the lysosome [10], [11]. The lysosome is the final repository of cargo taken up from the host serum for nutritional and immune evasion purposes. Trypanosomes are heme auxotroph. BS *T. brucei* obtain the cofactor by receptor-mediated endocytosis of the host haptoglobin/hemoglobin (Hb) complex and release of the heme in the lysosome [12], [13]. Also iron is acquired by endocytosis of a plasma protein. After internalisation of the host holo-transferrin [14], [15], iron is set free in the lysosome and apo-transferrin is proteolytically degraded [16]. Iron is required for DNA synthesis, antioxidant defence, mitochondrial FeS cluster biosynthesis, and, depending on the developmental stage, for mitochondrial respiration and alternative oxidase [17].

*T. brucei* lack catalase and selenocysteine-containing glutathione peroxidases. Hydroperoxide detoxification is achieved by 2-Cys-peroxiredoxins and non-selenium glutathione peroxidase (Px)-type enzymes which both obtain their reducing equivalents from the parasite-specific trypanothione/tryparedoxin system (for a recent review see [18]). Whereas the 2-Cys-peroxiredoxins use hydrogen peroxide as main substrate, the Px-type enzymes preferably detoxify lipid-derived hydroperoxides [19]. RNA interference studies targeting the cytosolic 2-Cys-peroxiredoxin or the three isoforms of the Px-type enzymes revealed that both types of peroxidases are essential [20], [21]. Proliferation of the Px-depleted BS parasites, however, can be fully restored by supplementing the culture medium with the vitamin E analogue Trolox as it is the case for GPx4-deficient mammalian cells [22]. This allowed us to clone cell lines that lack the individual genes [19]. The selective knockout of the mitochondrial *px III* gene results in parasites that display only a minor and transient growth retardation *in vitro* when compared to wild-type (WT) cells. In contrast, parasites lacking the cytosolic peroxidases (*px I–II* ^−/−^ cells) die within less than two hours after transfer into Trolox-free medium [19]. The relevance of Px III for parasite survival *in vivo* as well as the endogenous source of oxidants and the cell death mechanism of the *px I–II^−/−^* trypanosomes remained however elusive.

We here show that the mitochondrial Px III is fully dispensable for parasitism and identify a master role for the cytosolic trypanothione/tryparedoxin-dependent peroxidases in protecting the terminal lysosome. Our data revealed that, in the absence of Trolox, BS *px I–II^−/−^ T. brucei* undergo lysosomal disintegration, followed by damage of the mitochondrion - and likely other cellular membranes - and total cell lysis. The process is closely linked to the endocytic uptake of iron ions. The discovery of this unprecedented biological role of cytosolic thiol peroxidases is expected to have strong impact on respective studies in other human pathogens as well as the mammalian host.

## Results

### Lysis of the *px I–II^−/−^* bloodstream cells in the absence of Trolox is temperature-dependent

**Figure 1:**
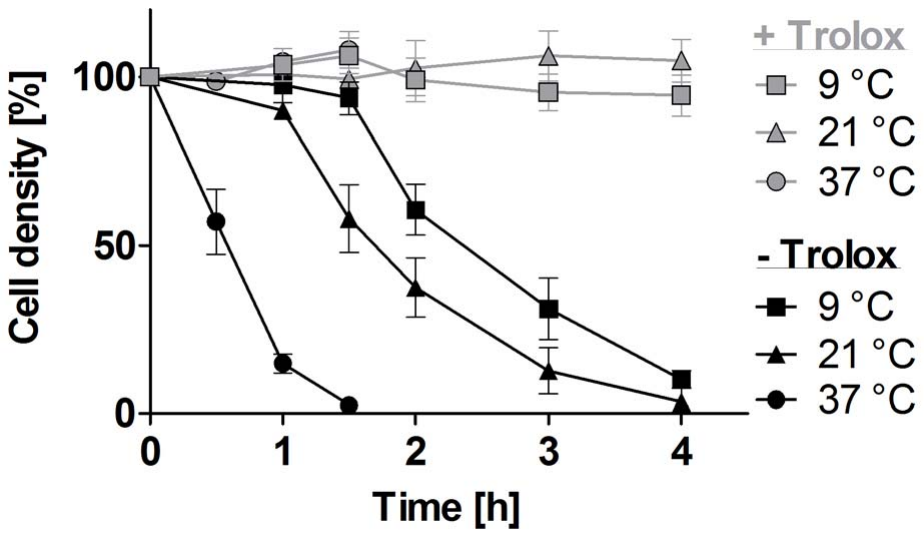
Lysis of the *px I–II* ^−/−^ BS cells is temperature-dependent. The *px I–II^−/−^* parasites were seeded at a density of 5–9×10^5^ cells/ml in standard HMI-9 medium (which contains 10% FCS) supplemented ±100 µM Trolox. The cells were incubated at 37°C, 21°C, and 9°C, respectively. At the indicated time points, living cells were counted. The values represent the mean ± SD of three independent experiments.

As a first step to elucidate the cellular processes responsible for the lethal phenotype of the *px I–II* ^−/−^ cells in the absence of Trolox, we followed the viability of the cells at different temperatures. After 90 min incubation at 37°C, the mutant parasites were completely lysed, whereas 60% and >90% of the cells were still viable when the parasites were kept at 21°C and 9°C, respectively (Figure 1). In contrast to the mammalian BS form, procyclic *px I–II* ^−/−^ cell lines were fully viable in the absence of Trolox (Figure S1). This may at least partially be due to the lower endocytosis rate in the insect form which is down-regulated approximately 10-fold compared to that of the BS form [23]. Because of the extremely rapid cell lysis observed at the normal culture temperature of 37°C, the uptake studies described in the following sections were conducted at room temperature (RT) to allow acquisition of reliable data. Endocytosis by BS *T. brucei* is highly sensitive to temperature [24]. To verify that under our conditions, cargo is still delivered to the lysosome, we followed the uptake of Alexa Fluor 488-conjugated dextran by living WT and *px I–II^−/−^* cells (the latter ones in the presence of Trolox) at different temperatures. Whereas after 10 min incubation at 37°C, all parasites displayed a discrete lysosomal staining, practically none of the cells was stained at 19°C and 9°C. After 2 h at 19°C, but not at 9°C, labeling of the cells was identical to that at 37°C in accordance with the dextran reaching the lysosomal compartment albeit at a much slower rate compared to 37°C (Figure S2).

### Lysosomal damage precedes lysis of the *px I–II^−/−^* cells

**Figure 2:**
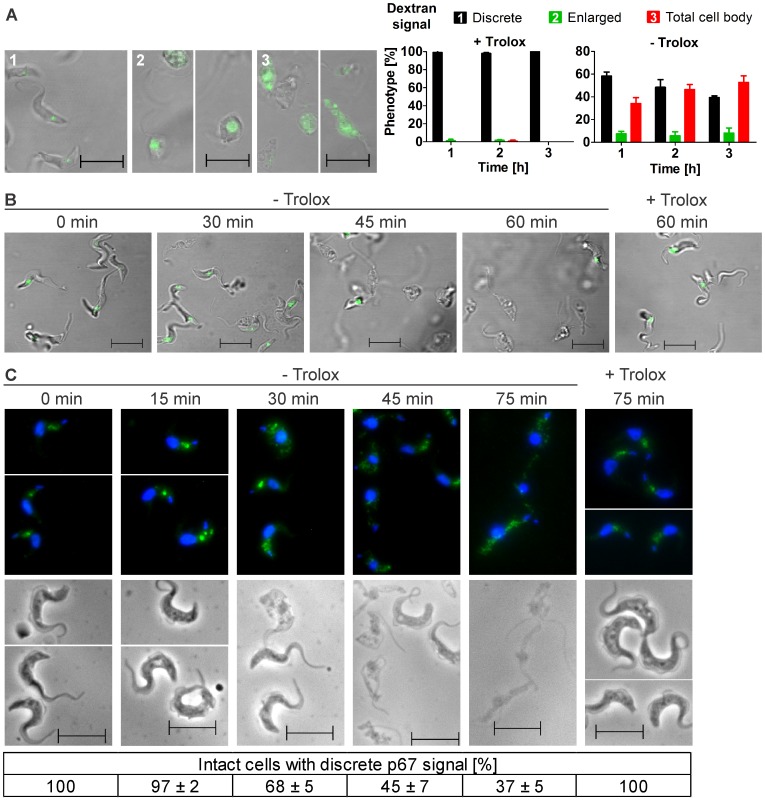
Withdrawal of Trolox results in morphological changes of the *px I–II* ^−/−^ BS cells. **A.** Living parasites were fed at 37°C with Alexa Fluor-488 conjugated dextran and then kept at RT in medium with (+) or without (−) 100 µM Trolox as outlined under Materials and Methods. On the left site, representative cells for the three different phenotypes observed in the remaining intact and fluorescent parasites are shown. The pictures were taken from cells incubated (1) 1 h, + Trolox, (2) 1 and 2 h, - Trolox, and (3) 2 h, - Trolox. On the right site, the quantitative analysis is provided. At each time point, ≥60 parasites were analyzed in three independent experiments and the mean ± SD was calculated. **B.** LysoTracker Green staining of living parasites incubated for the indicated times at 37°C in the presence or absence of Trolox. **C.** Immunofluorescence analysis of cells stained with antibodies against p67 (green) and DAPI (blue) to visualize nuclear (large dot) and kinetoplast (small dot) DNA (upper panel) and the corresponding phase contrast pictures (lower panel). At each time point, the p67 signal of at least 130 parasites was analyzed in each of three independent experiments and the mean ± SD was calculated (below). (Scale bar: 10 µm).

*Px I–II* ^−/−^ and WT cells were treated with Alexa Fluor 488-conjugated dextran and subjected to fluorescence microscopy. In the mutants kept with Trolox as in WT cells, the fluid-phase marker was discretely located in the post-nuclear region of the parasite consistent with lysosomal delivery (1 in Figure 2A). As expected, in the absence of Trolox, the *px I–II* ^−/−^ cells progressively lysed. Of the remaining fluorescent parasites, 40 to 60% displayed a signal that was spread over the whole cell body (3 in Figure 2A). The enlarged but still confined fluorescence observed in 5–10% of the cells (2 in Figure 2A) suggests that swelling of the organelle can occur as an intermediate step. This has been observed in parasites that were treated with protease inhibitors or human serum or upon ablation of p67, a lysosomal transmembrane glycoprotein [11], [25], [26]. Trypanolysis caused by apoL-I, crucial component of the trypanolytic factor present in human serum, involves the formation of anion-selective pores in the lysosomal membrane of the parasite, a process which is abolished by addition of 1 mM 4,4-diisothiocyanatostilbene-2,2-disulfonic acid (DIDS) to the culture medium [26], [27]. In the case of the *px I–II* ^−/−^ cells, 0.1 mM or 0.5 mM DIDS had no protective effect and 1 mM DIDS even proved to be lethal for both WT and mutant parasites (Figure S3). Thus, the loss of lysosomal integrity in the *px I–II* ^−/−^ cells does not appear to involve membrane pore formation.

To further dissect the role of the lysosome, the *px I–II* ^−/−^ cells were treated with LysoTracker, a fluorescent acidotropic reagent that traces acidic organelles in living cells and has previously been used to stain the parasite lysosome [28]. Cells that were kept at 37°C for up to 30 min without Trolox or for 60 min in the presence of the antioxidant showed a discrete lysosomal staining. In contrast, after 45 and 60 min in Trolox-free medium, many and virtually all, respectively, parasites had lost the fluorescent signal (Figure 2B). This finding confirmed that disintegration of the lysosomal compartment preceded cell lysis.

Antibodies against p67 typically stain a prominent vesicular compartment between the nuclear and mitochondrial (kinetoplast) DNA, although sometimes also multiple discrete vesicles in the same region are visualized [11], [29]. *Px I–II* ^−/−^ cells that were harvested up to 15 min after transfer into Trolox-free medium or kept in the presence of the antioxidant displayed a lysosomal p67 staining (Figure 2C). In contrast, when kept in Trolox-free medium, an increasing percentage of parasites lacked an intense and well defined signal. Many cells showed a dispersed staining which was reminiscent of parasites depleted of the Rab4 protein, a regulator of lysosomal trafficking [30]. These signals however were hardly to distinguish from the background fluorescence of cells treated only with the secondary antibody. Therefore, the quantitative analysis was based on cells with a vesicular p67 staining between the two DAPI signals. Taken together, the three different approaches strongly suggested that damage of the lysosomal membrane is a primary event caused by the absence of the cytosolic peroxidases.

### Exogenous iron speeds up lysis of the *px I–II^−/−^* cells

**Figure 3:**
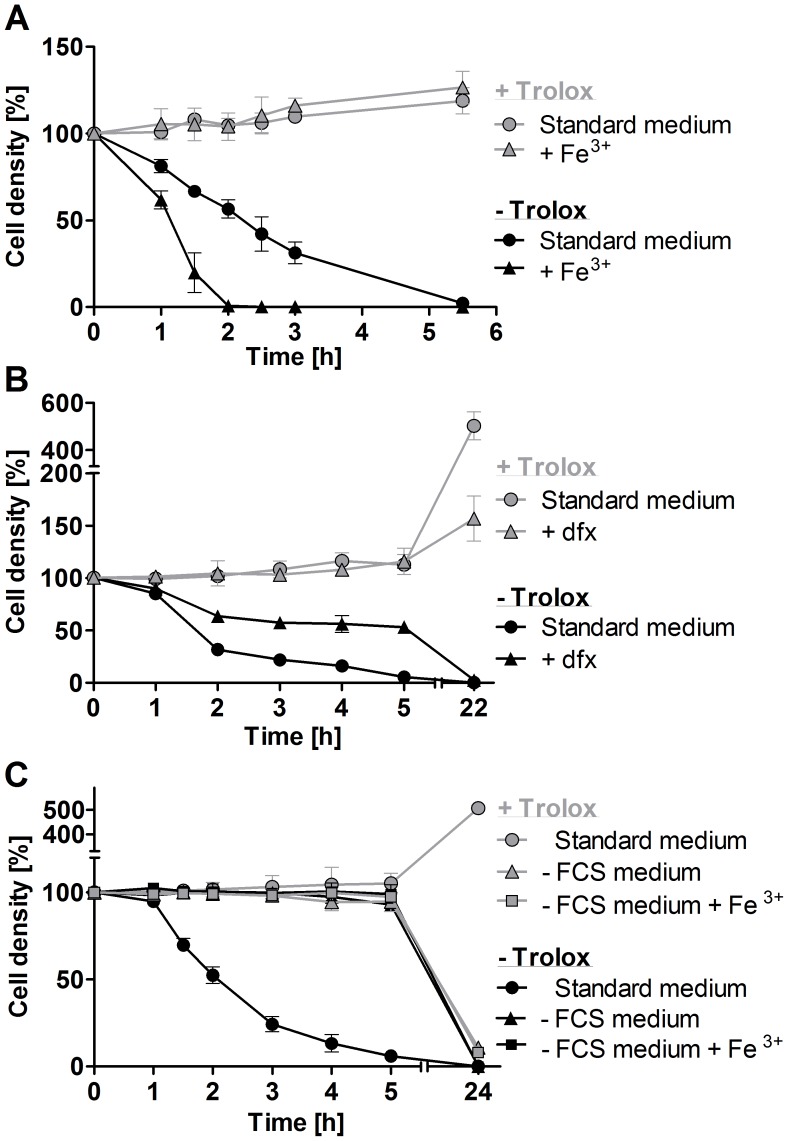
Exogenous iron promotes lysis of the *px I–II* ^−/−^ BS cells. **A.** The mutant parasites were incubated for up to 5.5°C in standard medium ±100 µM Trolox in the presence and absence of 100 µM Fe^3+^. WT cells behaved like the mutant cells in the presence of Trolox. **B.** The *px I–II^−/−^* cells were incubated for 5 h at 22°C and then cultured overnight at 37°C in medium ±100 µM Trolox in the presence and absence of 100 µM deferoxamine (dfx). WT behaved like the mutant cells in the presence of Trolox. **C.** The *px I–II^−/−^* cells were incubated for 5 h at 19°C and then cultured overnight at 37°C in medium ±10% FCS and/or 100 µM Trolox in the presence and absence of 100 µM Fe^3+^. The behavior of WT cells in the absence of FCS was identical to that of the mutant cells. At the different time points, living cells were counted. The values represent the mean ± SD of three independent experiments.

Supplementation of the medium with 100 µM iron chloride had no effect on the viability of the *px I–II* ^−/−^ cells provided the presence of Trolox. In the absence of the antioxidant, iron strongly accelerated cell lysis (Figure 3A). To further evaluate the role of iron, the parasites were treated with deferoxamine, a potent iron chelator that in mammalian cells is preferentially taken up by fluid-phase endocytosis [31]. Deferoxamine primarily prevents iron incorporation into newly synthesized proteins [32]. In Trolox-free medium, indeed the chelator slowed down cell lysis and thus protected the *px I–II* ^−/−^ cells (Figure 3B). In the presence of Trolox, deferoxamine had no effect on the short-term viability of the mutant cells but inhibited the long-term cell proliferation, as it has previously been shown for WT parasites [32], [33]. Neither iron nor deferoxamine affected the short term viability of WT cells independent of the presence or absence of Trolox (not shown).

### A serum component contributes to cell lysis

BS *T. brucei* are cultured in HMI-9 medium which contains 10% of FCS. In accordance with our previous observations, *px I–II* ^−/−^ cells kept in this standard medium rapidly died, and after 2 h, the culture displayed only 50% of the starting cell density (Figure 3C). In medium lacking FCS, however, the mutant parasites remained viable and, remarkably, were insensitive towards exogenous iron, even in Trolox-free medium. Overnight cultivation in medium without FCS resulted in complete cell death due to the lack of essential nutrients and growth factors. In conclusion, the iron-induced cell lysis clearly required the presence of (a component of) FCS. BS *T. brucei* acquire heme by receptor mediated endocytosis of the haptoglobin-Hb complex [12]. In the absence of Trolox, supplementing the medium with 1 mg/ml of Hb resulted in a minor, but detectable, acceleration of cell lysis (Figure S4). This only moderate effect may at least partially be due to the fact that in the serum, haptoglobin is already essentially saturated with Hb.

### Transferrin stimulates cell lysis

**Figure 4:**
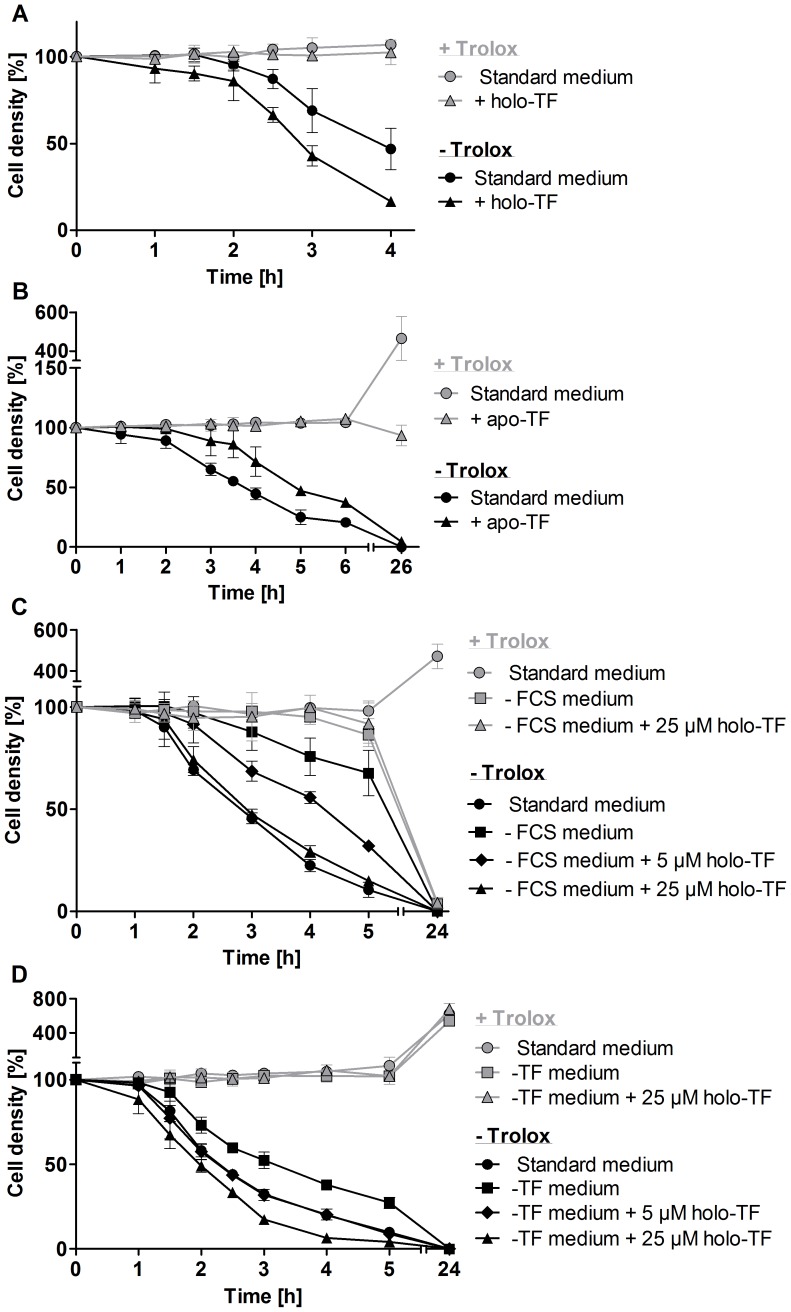
Holo-transferrin induces, whereas apo-transferrin slows down lysis of the *px I–II* ^−/−^ BS parasites. The mutant cells were incubated **A.** for 4 h at RT in standard medium ±100 µM Trolox in the presence and absence of 25 µM holo-transferrin (holo-TF), **B.** for 6 h at RT and then cultured overnight at 37°C in medium ±100 µM Trolox in the presence and absence of 320 µM apo-transferrin (apo-TF), **C.** for 5 h at RT followed by overnight cultivation at 37°C in standard medium as well as in FCS-free medium ±100 µM Trolox in the presence and absence of 5 µM and 25 µM holo-TF, and **D.** for 5 h at 20°C in standard medium as well as in transferrin-depleted medium (-TF medium) ±100 µM Trolox in the presence and absence of 5 µM and 25 µM holo-TF. The values represent the mean ± SD of three independent experiments.

FCS contains about 25 µM transferrin and the iron saturation of transferrin ranges from 55 to 92% [34]. To mimic these conditions, the medium was supplemented with 25 µM holo-transferrin. In the absence of Trolox, this treatment stimulated lysis of the *px I–II* ^−/−^ cells (Figure 4A). In contrast, apo-transferrin slowed down cell lysis in the absence of Trolox (Figure 4B). Parasites incubated overnight with apo-transferrin in medium containing Trolox remained viable but did not proliferate. Competition between holo- and apo-transferrin for the parasite transferrin receptor results in reduced iron uptake [14]. This should directly affect the synthesis of DNA precursors by the iron-dependent ribonucleotide reductase. Incubation of the mutant parasites with holo-transferrin in medium lacking both FCS and Trolox induced the cell lysis (Figure 4C). However, a 10-fold higher concentration of holo-transferrin (25 µM) was required for an effect comparable to that observed in the presence of 10% FCS. This suggests that in the absence of FCS, the overall metabolism of the parasite is affected and/or another serum component contributes to the lethal phenotype. To get a deeper insight in the mechanism, we prepared transferrin-depleted medium. Purified antibodies against bovine transferrin were covalently linked to sepharose and HMI-9 medium was chromatographed on this matrix. Western blot analysis confirmed the successful removal of transferrin from the medium (Figure S5). As expected, in the presence of Trolox, the *px I–II^−/−^* cells did not show any lysis in the transferrin-depleted medium with or without supplementation by 25 µM transferrin (Figure 4D). However, in the absence of Trolox, the *px I–II* ^−/−^ cells displayed lysis. This may be due to the uptake of Hb, which occurs via endocytosis of the haptoglobin/Hb complex, again resulting in lysosomal iron [26]. In addition, we cannot rule out that the medium still contained residual transferrin that was not detected in the Western blot. Even when assuming 99% depletion, the remaining transferrin may still be efficiently internalized due to the high affinity of the parasite transferrin receptor [14]. The contribution of transferrin to cell lysis could be clearly demonstrated: Supplementing the transferrin-depleted medium with transferrin accelerated cell lysis. Upon overnight cultivation in Trolox-supplemented transferrin-depleted medium, the mutant parasites proliferated, again indicating that the medium contained an iron source. This is in accordance with previous work showing that WT *T. brucei* can grow in transferrin-depleted medium [35]. Taken together, the data suggest that transferrin as well as Hb contribute to the lethal phenotype of the *px I–II^−/−^* cells in the absence of the exogenous antioxidant, both resulting in the generation of free lysosomal iron.

### Mitochondrial damage is a secondary event

**Figure 5:**
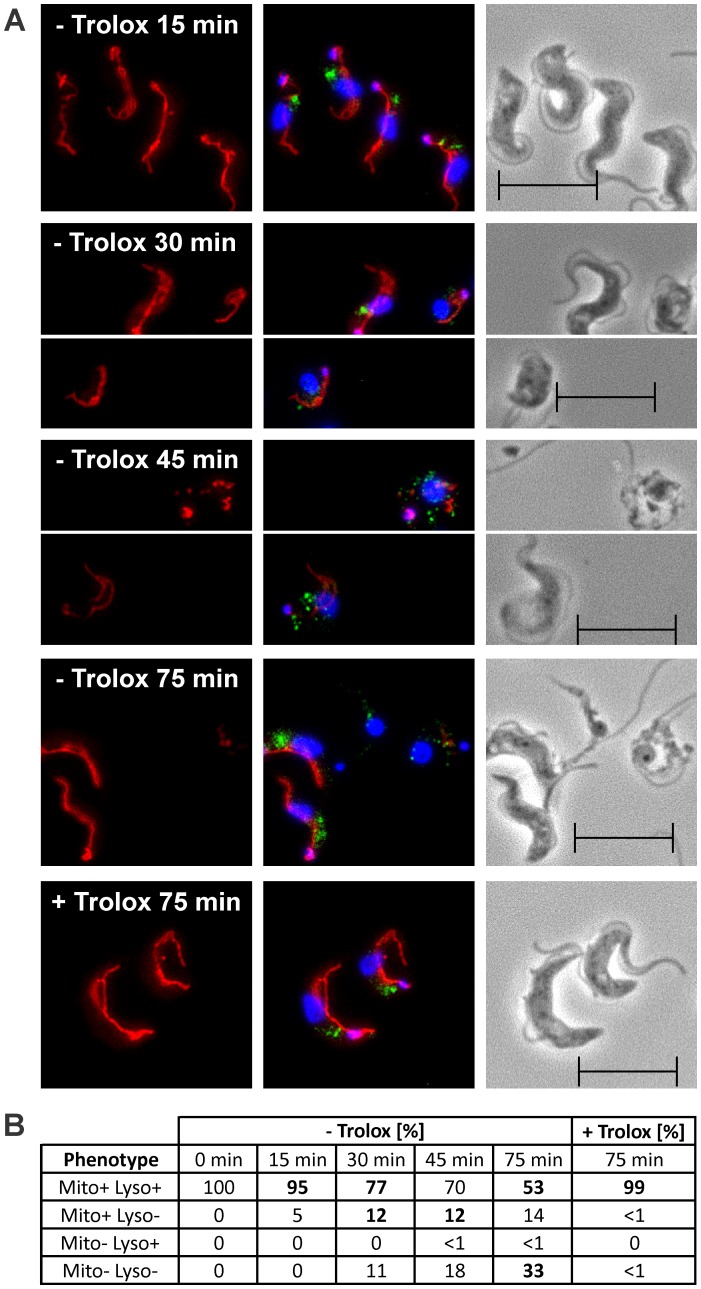
Lysosomal disintegration precedes damage of the mitochondrion. The *px I–II* ^−/−^ BS cells were kept for the indicated times at 37°C in medium ± Trolox and then subjected to immunofluorescence analysis. **A.** MitoTracker staining (left), overlay of the signals for MitoTracker (red), p67 (green), and DAPI (blue) (middle), and phase contrast images (right). **B.** Quantitative analysis of the staining pattern of the cells (for details, see text). The phenotypes visible in the respective pictures in (**A**) are highlighted by bold numbers. For each time point, at least 194 cells were inspected. The data are representative of two independent sets of experiments giving very similar results. (Scale bar: 10 µm).

The *px I–II* ^−/−^ cells were transferred from Trolox-supplemented into medium ± Trolox and after different time points subjected to immunofluorescence microscopy with MitoTracker, p67 antibodies, and DAPI (Figure 5A). The parasites were subdivided into four groups that displayed a) both MitoTracker and discrete p67 staining; b) MitoTracker staining, but no discrete p67 signal; c) no MitoTracker staining, but discrete p67 signal; and d) neither MitoTracker nor p67 staining (Figure 5B). *Px I–II* ^−/−^ cells kept for up to 15 min in Trolox-free medium and those in the presence of the antioxidant displayed perfect mitochondrial and lysosomal signals. In contrast, prolonged incubation of the *px I–II* ^−/−^ parasites in Trolox-free medium resulted in the progressive formation of cells that lacked both signals. About 12% of the cells showed MitoTracker staining but no discrete p67 signal. The reverse phenotype, namely parasites that lacked the MitoTracker staining but had a discrete p67 signal, was practically not observed. Thus, lysosomal disintegration precedes the damage of the mitochondrion.

### *T. brucei* lacking the mitochondrial Px III display moderately attenuated virulence

BS *T. brucei* in which the gene encoding the mitochondrial Px III has been knocked out display an only minor and transient proliferation defect *in vitro* [19]. It remained however elusive whether the *px III* ^−/−^ parasites adapted to *in vitro* growth with or without Trolox are also able to cope with the more hostile environment in the mammalian host.

**Figure 6:**
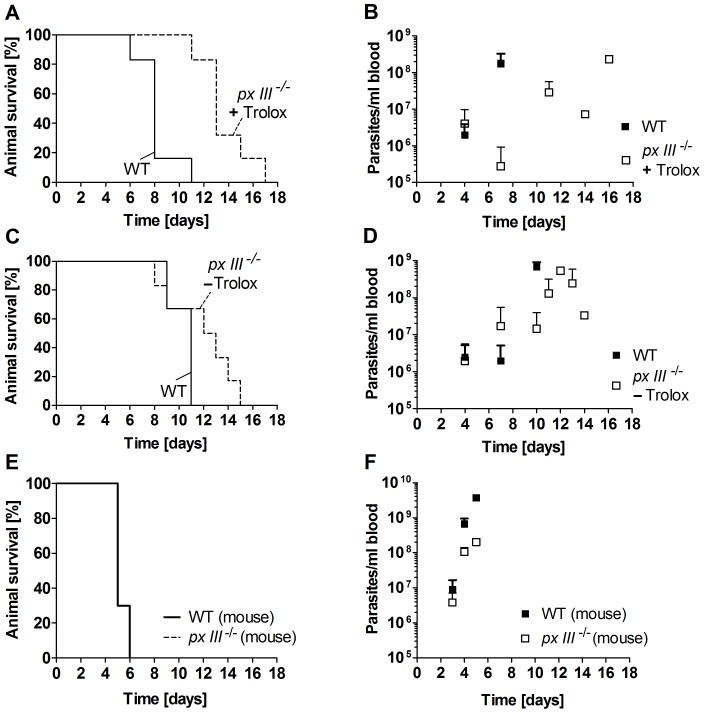
Infectivity of *px III* ^−/−^ and WT *T. brucei*. **A.** and **B.** Female BALB/cJ mice (n = 6/group) were infected intraperitoneally with 10^4^ WT parasites or *px III* ^−/−^ cells that, prior to infection, had been cultivated in medium supplemented with 100 µM Trolox, and **C.** and **D.** in medium without Trolox. **E.** and **F.** Mice (n = 3/group) were inoculated with *px III* ^−/−^ and WT parasites isolated from infected animals (for details see Materials and Methods). **A.**, **C.**, and **E.** Kaplan-Meyer plots for animal survival, **B.**, **D.**, and **F.** Average parasite load in blood samples taken from the animals at the indicated times. All data are the mean value ± SD.

In the first series of *in vivo* experiments, mice were infected with *px III* ^−/−^ cells that had been cultured in the presence of 100 µM Trolox. As depicted in Figure 6A, the animals showed a 5-days extended medium survival time compared to mice infected with WT parasites. These differences could not be ascribed to an impaired initial infectivity, since at day 4 post-infection, all animals from both cohorts were infected and displayed a very similar average parasite burden (Figure 6B). The delayed disease progression of the *px III* ^−/−^ group (p-value of 0.017 for a log rank test [36]) was however in agreement with the overall slower *in vivo* proliferation of the mutant parasites compared to the WT strain.

To elucidate if growth in the presence of the antioxidant was responsible for the delayed phenotype, in the second series of experiments mice were infected with *px III* ^−/−^ cells that had been cultured in the absence of Trolox. Although the mean survival time of the animals infected with WT and mutant parasites was not significantly different, a log rank test [36] gave a p-value of 0.046 again suggesting a slightly delayed lethality for the animals infected with the *px III^−/−^ T. brucei* (Figure 6C). The survival profiles of mice infected with *px III* ^−/−^ cells were comparable independently if the parasites had been cultured with or without Trolox prior to infection (Figures 6A and C). Only a slightly faster overall development of parasitemia was observed for the *px III* ^−/−^ -Trolox group compared to the +Trolox group (Figures 6B and D). Whether this is indicative of an adaptive mechanism to overcome the lack of the mitochondrial peroxidase in the absence of Trolox complementation remains to be investigated.

Passage through animal has been shown to increase the virulence of parasites maintained *in vitro* [37]. To assess whether *px III* ^−/−^ trypanosomes are capable to recover the virulence of the parental cell line, mice were infected with *px III* ^−/−^ and WT parasites isolated from infected animals. In both groups, the disease developed very rapidly resulting in an identical profile of animal death (Figure 6E). As observed in the experiments described above, parasitemia appeared to develop more slowly in mice infected with the *px III* ^−/−^ cells compared to the WT parasites (Figure 6F). Taken together, and irrespective of the reasons that cause their initially attenuated *in vivo* phenotype, the *px III* ^−/−^ parasites fully restored their virulence. Thus, the mitochondrial peroxidase is clearly not essential for *T. brucei* to infect the mammalian host.

## Discussion

African trypanosomes express three virtually identical Px-type proteins in the cytosol (Px I–II) and mitochondrion (Px III). The closest related enzyme in higher organisms is GPx4 [3], [38]. Both types of enzymes have in common that they are monomeric proteins, prefer lipid-derived hydroperoxides as substrates, and their physiological functions can be replaced by α-tocopherol or Trolox but not by water-soluble antioxidants [19], [22], [39]. In contrast to GPx4, the parasite Px I–III do not contain a selenocysteine but a cysteine residue in the active site and obtain their reducing equivalents from the kinetoplast-specific trypanothione/tryparedoxin system, not from glutathione [21], [40]. In mammals, the cytosolic GPx4 is the only known glutathione peroxidase that is essential. The inducible inactivation of the cytosolic GPx4 in mice revealed that the enzyme counteracts the activity of 12/15 lipoxygenase which catalyzes hydroperoxide formation in membranes and triggers an apoptosis-inducing factor-mediated cell death [22]. Intriguingly, the mechanism of cell death remained unclear since the canonical markers of programmed cell death (e.g. caspase 3 activation, phosphatidylserine exposure) were not detected. Only disruption of the mitochondrial membrane potential was observed as a late event upon induction of the GPx4 knockout [22].

Here we show that the lethal phenotype of BS *px I–II* ^−/−^ trypanosomes originates from the damage of their lysosome. *Px I–II* ^−/−^ cells deprived of Trolox and treated with Alexa Fluor-conjugated dextran became completely fluorescent with time; and cells fed with LysoTracker lost the fluorescent signal totally. The enlarged but still confined fluorescent signal observed in a fraction of the dextran-fed *px I–II* ^−/−^ parasites indicates that lysosomal swelling may precede rupture of the organelle. Our data did not support a specific pore formation in the lysosomal membrane as it is the case in parasites treated with the trypanolytic factor component apoL-I [26], [27]. Probably a reduced rate of export and/or recycling of (the damaged) membrane and content contributes to the enlargement of the lysosome, a mechanism discussed in the context of p67-ablated parasites [11]. Accordingly, the immunofluorescence analysis of *px I–II* ^−/−^ cells with p67 as lysosomal marker showed a progressive loss of the discrete organelle signal. All these findings strongly suggest that cell death starts with disintegration of the lysosome and likely evolves to massive membrane damages, as shown here for the mitochondrion. The absence of these cytosolic peroxidases probably affects the integrity of all (sub)cellular membranes. The Px-type enzymes use trypanothione as substrate suggesting that a decrease of the low molecular weight thiol should cause a similar phenotype. Indeed, the ultrastructural analysis of *T. brucei* depleted of trypanothione synthetase indicates membrane damage at several organelles [41]. However, lowering the trypanothione level will affect also the peroxiredoxin-type peroxidase and many other redox pathways and therefore the overall cellular redox state.

Lysosomes are oxidizing rather than reducing compartments [42] and therefore should require the presence of effective antioxidant systems. Dietary vitamin E has been reported to result in increased α-tocopherol levels in the lysosomal fractions and to prevent lysosomal release [43]. The full rescue of the lethal phenotype of the *px I–II* ^−/−^ parasites by Trolox strongly suggests that one physiological role of the cytosolic peroxidases is protection of the lysosomal membrane from peroxidation.

Both free iron and heme are known to react with hydrogen peroxide to generate highly oxidizing species that are capable of initiating lipid peroxidation [13], [44]. Indeed, supplementing Trolox-free medium with iron or holo-transferrin accelerated lysis of the *px I–II* ^−/−^ cells whereas the removal of FCS or addition of apo-transferrin slowed down trypanolysis. In addition, the use of transferrin-depleted medium suggests that also iron derived from the uptake and degradation of Hb contributes to the lethal phenotype of the *px I–II^−/−^* cells in the absence of Trolox. The lysosomal iron probably induces damage of the organelle membrane by inducing a Fenton-like reaction as described previously [13]. In line with a key role of iron in lysosomal damage, treatment of *px I–II* ^−/−^ cells with the iron chelator deferoxamine [32], [33] increased their short-term viability in Trolox-free medium. In human epithelial cells and lysosome-rich murine macrophage-like J774 cells, deferoxamine localizes almost exclusively within these organelles [31], [45]. Also for *T. brucei*, the lysosome has been suggested as an iron storage organelle [46] and a mucolipin 1 orthologue has been implicated in iron transport into the cytosol [47]. Intralysosomal iron can powerfully synergize oxidant-induced cellular damage. The steps associated with cell collapse in different mammalian cell lines were not fully understood [31], [48] although it was clear that lysosomal disruption entailed cell death [6].

In BS *px I–II^−/−^ T. brucei*, the cellular damage is apparently linked to endocytosis. This is supported by several observations. In the absence of FCS, the *px I–II^−/−^* cells do not require Trolox for short-term viability and are even insensitive towards exogenous iron. This would not be expected if the cellular damage started at other membranes such as e. g. that of the glycosomes or mitochondrion or the plasma membrane. Disintegration of the cell membrane as primary event could be ruled out also by the fact that after feeding with fluorescent dextran and transfer into Trolox-free medium, a large proportion of the *px I–II^−/−^* cells became completely stained. Since in a variety of other cell types, the mitochondrion is the origin of endogenous oxidative stress we studied the damage of the lysosome and mitochondrion in more detail. In the *px I–II* ^−/−^ parasites, mitochondrial damage occurs but follows the disintegration of the lysosome. The order of events is thus opposite to that in cells with injured mitochondria where externalization of cardiolipin to the outer mitochondrial membrane acts as an elimination signal for the mitochondrion by autophagy [49].

Notably, any treatment, such as with exogenous iron, Hb or transferrin affected the viability of the *px I–II* ^−/−^ cells only in the absence of Trolox. All of these compounds enter the parasite via the endocytic pathway suggesting that the extremely fast lethal phenotype of BS trypanosomes that lack the cytosolic peroxidases is linked to their high endocytic rate. Taken together, disintegration of other intracellular membranes and the plasma membrane appears to be a secondary event or is at least much slower than lysosomal damage.

Parasites inhabiting the insect vector harbor a fully developed mitochondrion rich in cytochromes and Krebs cycle enzymes, many of which require iron or iron/sulfur complexes as cofactors [50]. Strikingly, in the insect stage, the cytosolic Px I–II proved to be entirely dispensable under culture conditions. One reason may be the lower endocytic activity of the procyclic cells compared to BS parasites [23]. In addition, procyclic *T. brucei* do not express a transferrin receptor [51] but can take up iron via specific transporters [52] or extract it from internalized hemin as it is the case in *Leishmania infantum* [53]. *L. amazonensis* has been shown to express a heme transporter (LHR1) in both the plasma membrane and acidic endocytic compartments; and highly syntenic, close homologs are present in *T. brucei* [54]. The direct delivery to the cytosol and/or the rapid transport from the lysosome into the mitochondrion probably precludes lysosomal iron accumulation in the procyclic cells and thus renders Px I–II not necessary for protection of this organelle towards iron-mediated lipid peroxidation. On the other hand, these stage-specific differences highlight the biological role of the cytosolic peroxidases at the interface between iron homeostasis and protection against iron-induced and lipid-derived oxidative stress in the pathogenic stage of the parasites.

BS *T. brucei* lack an active respiratory chain [7] and, although obligate, mitochondrial iron-sulfur cluster biosynthesis is much lower compared to the insect stage [9], [17]. These may be main reasons why BS parasites in which the mitochondrial *px III* gene has been knocked out do not display any strong proliferation phenotype [19]. The *in vivo* data presented here revealed that the Px III is not essential for infectivity and survival although mutant parasites grown *in vitro* prior to infection displayed a slightly delayed proliferation and disease development compared to WT pathogens. The cytosolic isoenzymes are probably sufficient for protecting the membranes of the metabolically repressed mitochondrion of BS parasites. Despite the fact that the virulence of the *px III^−/−^* cells was fully restored after a single passage through mouse, again the parasitemia developed more slowly than in animals infected with the parental cell line. This may suggest a permanent, albeit minor, impairment by the lack of a Px III-dependent function. Remarkably, one week after infection with the *px III* ^−/−^ parasites cultured in the presence or absence of Trolox, in four and two, respectively, of the six mice, the blood parasitemia had dropped to undetectable levels. This was not the case in any of the 12 animals infected with WT parasites and may be a consequence of the host innate immune defense. In the early stage of African trypanosomiasis, one of the hallmarks is the activation of the macrophage/monocyte system (for reviews see [55], [56]). As a result, reactive nitrogen and oxygen species are released and have cytotoxic and cytostatic effects on trypanosomes. Notably, IFN-γ and TNF-α, two cytokines participating actively in parasite control during the early stage of the infection [56], peak around the first week post-infection [57]. One may thus speculate that the mitochondrial peroxidase could contribute to the parasite defense towards exogenous oxidative stresses.

As shown here for African trypanosomes, cytosolic glutathione peroxidase-type enzymes are responsible for protecting the lysosome from oxidative membrane damage. To our knowledge, this is the first report demonstrating such function for cytosolic thiol peroxidases. The underlying molecular mechanism is not yet known and we can only speculate. Peroxidized phospholipids formed in the inner leaflet of the organelle membrane may be exchanged with lipids in the outer leaflet to allow the subsequent repair by the cytosolic peroxidases. In addition, it remains elusive if this lipid exchange would be a spontaneous or catalyzed process. At least for the plasma membrane of different *Leishmania* species, a phospholipid scramblase activity has been described [58]. In addition, cardiolipin externalization to the outer mitochondrial membrane has been demonstrated recently [49]. Although not yet investigated in detail, it is worth to note that other human pathogens such as *Trichomonas*, *Toxoplasma*, and *Entamoeba* can acquire iron via endocytic uptake of host Fe-binding proteins such as transferrin, lactoferrin, and ferritin (for reviews see [59], [60]) and the genomes of *T. vaginalis* and *T. gondii* encode Px homologues. Future work by others may reveal if our findings are extensible to these pathogens and the mammalian GPx4.

## Materials and Methods

### Materials

*Px I–II* ^−/−^ BS *T. brucei* and polyclonal rabbit antibodies against Px were generated previously [19]. Bovine holo-transferrin, 10,000 Da dextran conjugated to Alexa Fluor 488, LysoTracker Green DND-26, DIDS, and MitoTracker Red CMXRos were purchased from Life Technologies. FeCl_3_, deferoxamine mesylate, bovine Hb, bovine apo-transferrin, (±)-6-hydroxy-2,5,7,8-tetramethylchromane-2-carboxylic acid (Trolox), DAPI, and phleomycin were from Sigma. FCS was from Biochrome. Affinity-purified bovine transferrin antibodies were purchased from Bethyl Laboratories Inc. The monoclonal mouse anti-p67 antibody was a kind gift of Dr. James D. Bangs, Buffalo and the polyclonal rabbit anti-aldolase antibody was kindly provided by Dr. Christine Clayton, Heidelberg.

### Cultivation of bloodstream *T. brucei* and phenotypic analyses

WT and mutant BS *T. brucei* (449 cells from Lister strain 427) were cultivated in HMI-9 medium [61] without serum plus, supplemented with 36 mM NaHCO_3_, 50 U/ml penicillin, 50 µg/ml streptomycin, and 0.2 µg/ml phleomycin. This medium contains 10% FCS ( = standard medium). Thus, unless otherwise stated, all experiments were performed in the presence of 10% FCS. The *px I–II* ^−/−^ cells were grown in the presence of 100 µM Trolox. Parasites were harvested and studied at a density of 3–9×10^5^ cells/ml in medium ± Trolox, ± FCS and supplemented with FeCl_3_, deferoxamine, Hb, holo-transferrin, apo-transferrin, and DIDS, respectively. Viable cells with normal morphology were counted in a Neubauer chamber and the mean ± SD of three independent experiments was calculated. Statistical analyses were performed with Prism (GraphPad) and Microsoft (Excel) software and evaluated by paired two-tailed Student's t-test. Differences were considered to be significant when the p-value was ≤0.05.

### Generation of transferrin-depleted medium

The procedure for preparing the transferrin-depleted HMI-9 medium was adapted from those described by Schell et al. [62] and Ekblom et al. [63]. Affinity-purified bovine transferrin antibodies (12 mg) were coupled to 8 ml of CNBr-activated Sepharose 4B (GE Healthcare Life Sciences) according to the manufacturer's instructions yielding 11 mg covalently bound protein. The column was equilibrated with FCS-free HMI-9 medium. Standard medium was applied at a flow rate of 0.2 ml/min and 5 ml fractions were collected. After each pass, the column was washed with PBS, 8 M urea in PBS to remove bound transferrin, again with PBS, and re-equilibrated in FCS-free medium. The successful removal of transferrin was confirmed by Western blot analysis. The first pass yielded three, all subsequent passes, two transferrin-free fractions. Fractions devoid of transferrin were pooled yielding 45 ml of transferrin-depleted HMI-9 medium.

### Cloning and cultivation of procyclic *px I–II* ^−/−^ *T. brucei*

To replace the *px I* and *II* genes in the insect stage, procyclic *T. brucei* 449 cells were transfected with the vectors pHD1747-KO*pxI*(*I*–*II*) and pHD1748-KO*pxI*(*I*–*II*) originally generated for the respective work in BS cells [19]. The successful replacement of both alleles by a puromycin and blasticidin resistance gene, respectively, was verified by PCR analyses (Figure S1A, Table S1). Procyclic cells were grown at 27°C in MEM-Pros medium as described previously [21].

### Western blot analysis

2×10^6^ Procyclic *T. brucei* cells were harvested, boiled for 10 min in 1× sample buffer containing 2-mercaptoethanol, and subjected to SDS-PAGE (12% gel). In the case of the transferrin-depleted medium, 0.5 µl of the standard medium (corresponding to 100 ng transferrin) and of the fractions collected from the immuno column were applied. After blotting, the PVDF membranes were reacted with the primary antibody against Px (1∶2000), aldolase (1∶40000), and transferrin (1∶1000), respectively, followed by the goat anti-rabbit and rabbit anti-sheep immunoglobulin G conjugated to horseradish peroxidase antibodies (1∶10000, Santa Cruz Biotechnology), respectively, and developed by the SuperSignal West Pico Chemiluminescent Substrate kit (Pierce).

### Life cell imaging

The parasites were kept for 30 min at 37°C in medium ± Trolox, harvested, resuspended in 50 µl of the respective medium containing 2.5 mg/ml Alexa Fluor 488-conjugated dextran, and incubated for 10 min. The cells were washed twice with medium and life cell imaging was performed as previously described [19]. For LysoTracker staining, the cells were incubated for various times at 37°C in medium ± Trolox. Thirty minutes prior to analysis, 5 µM LysoTracker was added. After twice washing with medium, the slides were examined using a Carl Zeiss LSM 510 confocal microscope and the LSM 510 software (Zeiss, Jena). The data presented are the mean ± SD of three independent experiments.

### Immunofluorescence microscopy

About 2×10^6^ cells from logarithmic growth phase were harvested and incubated in medium ± Trolox at 37°C for different times. For MitoTracker staining, cells were incubated in medium + Trolox containing 0.12 µM MitoTracker for 15 min at 37°C, washed with PBS, and incubated in medium + Trolox for 30 min at 37°C. After washing with PBS, cells were fixed in 4% paraformaldehyde in PBS for 20 min at RT, transferred to 8-well poly-L-lysine slides (BD Falcon), and allowed to settle down for 1 h at RT or to untreated slides and incubated overnight at 4°C. The cells were permeabilized with 0.2% Triton X-100 (v/v in PBS) for 20 min at RT and washed twice with PBS. Subsequently, the slides were treated with 0.5% gelatine in PBS for 20 min, incubated for 1 h at RT with the p67 antibody (1∶800 in 0.5% gelatine in PBS), and washed with PBS. After 1 h incubation with goat anti-mouse antibodies coupled to Alexa Fluor 488 (Molecular Probes, 1∶10,000) and PBS washing, the DNA was stained with 500 ng/ml DAPI in PBS for 15 min at RT. The cells were mounted and examined under a Carl Zeiss Axiovert 200 M microscope equipped with an AxioCam MRm digital camera using the AxioVision program (Zeiss, Jena). The quantitative analysis was based on cells that displayed a single DAPI signal for each nuclear and kinetoplast DNA. The data are presented as mean ± SD of three independent experiments.

### Ethics statement

The animal protocols used in this work were evaluated and approved by the Animal Use and Ethic Committee (CEUA) of the Institut Pasteur Montevideo (Protocol 2009_1_3284). They are in accordance with FELASA guidelines and the National law for Laboratory Animal Experimentation (Law no. 18.611).

### *In vivo* experiments

Thirty 6–8 weeks old Balb/cJ female mice were bred at the SPF animal facility of the Transgenic and Experimental Animal Unit of the Institut Pasteur de Montevideo (IPMon). They were housed in individual ventilated cages with negative pressure (Sealsafe rack, Tecniplast, Milano, Italy) in controlled environment at 20±1°C with a relative humidity of 40–60% in a 14/10 light-dark cycle. Food and water were administered *ad libitum*. The animals received a single intraperitoneal injection of 10^4^ parasites harvested in exponential growth phase and suspended in 0.3 ml fresh medium. The following groups were studied: animals infected with WT parasites grown *in vitro* or isolated from an infected mouse, animals infected with *px III* ^−/−^ parasites cultured for one week in medium ±100 µM Trolox or isolated from a mouse which had been infected with parasites grown *in vitro* in the absence of Trolox. The parasites isolated from an infected animal were enriched. Briefly, 5–10 µl of anti-coagulated blood subjected to hypotonic lysis of red blood cells was added to one well in a 24-well culture plate containing 1 ml medium and incubated at 37°C and 5% CO_2_. Proliferating parasites were transferred to culture flasks containing 5–10 ml medium and cultivated for 10 to 16 days followed by cryo-preservation. At least four days prior to infection, the parasites were thawed and grown to exponential phase. The health status and survival of infected animals were monitored daily. Parasitemia levels in mice were determined regularly in blood samples (≤50 µl) taken from the submandibular vein. Blood was collected in a tube containing tri-potassium ethylenediamine tetra-acetic acid (K3EDTA) anticoagulant at a blood∶K3EDTA ratio of 20∶1. After thorough homogenization, an aliquot was diluted 1∶20 in a hypotonic solution (BD Pharm Lyse) to lyse red blood cells, incubated for 2 min at RT and diluted in PBS-1% (w/v) glucose for cell counting. The minimum parasite density detectable by this method is about 2.5×10^4^ cells/ml. Mice showing an impaired health status and/or a parasite load of ≥10^8^ cells/ml blood were euthanized. For the data of the Kaplan-Meier survival plots, log rank tests [36] were performed and the mean ± SD was calculated for the average parasite loads.

**The genes studied in this work are:**

*px I*: Tb427.07.1120

*px II*: Tb427.07.1130

*px III*: Tb427.07.1140

(TriTrypDB: http://www.tritrypdb.org/tritrypdb/)

## Supporting Information

Figure S1 **Generation and phenotypic analysis of procyclic** ***px I–II^−/−^ T. brucei*** **.** **A.** Upper part. Genomic *px* locus. The *px I–II* genes were replaced by transfecting the parasites with constructs containing a puromycin and blasticidin resistance gene, respectively, flanked by 5′ XhoI/3′ HindIII and 5′ PstI/3′ NotI restriction sites 5′ and 3′ of the resistance gene, respectively, generated previously [19]. Middle part. To verify the specific removal of the *px I–II* alleles and correct insertion of the resistance genes after the two consecutive transfections, genomic DNA of two *px I–II^−/−^* cell lines (clones A and B) and WT parasites was subjected to PCR with different primer pairs (*pac*: P1 and P3, *bla*: P2 and P3, *px I*: P4 and P5, *px II*: P6 and P5, and *px III*: P7 and P8). Lower part. Western blot analysis of the two *px I–II^−/−^* clones and WT cells against Px and aldolase (AL) as loading control. The remaining comparably weak band corresponds to the mitochondrial Px III. **B.** Proliferation of the *px I–II* ^−/−^ cell lines (clone A and B) and WT cells in the presence (+) and absence (–) of 100 µM Trolox. The data are representative of three independent experiments giving identical results.
(TIF)

Figure S2 **Temperature-dependent uptake of fluorescent dextran by WT and** ***px I–II^−/−^*** **BS parasites.** Living cells were incubated in standard medium with 2.5 mg/ml Alexa Fluor-488 conjugated dextran at 37°C, 19°C, and 9°C for 10 min and 2 h. In the case of the *px I–II^−/−^* cells, the medium was supplemented with 100 µM Trolox. The major phenotype of the respective cell populations is displayed. At 37°C, already after 10 min, the whole cell population displayed lysosomal staining. At 19°C, after 10 min practically none of the cells showed a fluorescent signal, but after 2 h, the picture was indistinguishable from that at 37°C. At 9°C, no significant staining was observed. No difference was noticed between WT and mutant parasites. (Scale bar: 10 µm).
(TIF)

Figure S3 **DIDS does not protect the** ***px I–II*** **^−/−^ BS cells from lysis.** The mutant cells were incubated in standard medium ±100 µM Trolox containing none, 0.1, and 0.5 mM DIDS. The data represent the mean ± SD of three independent experiments.
(TIF)

Figure S4 **Supplementing the medium with hemoglobin slightly induces lysis of the** ***px I–II*** **^−/−^ BS parasites.** Cells were cultured in standard medium in the presence and absence of 100 µM Trolox and 16 µM hemoglobin at 21°C and subsequently incubated overnight at 37°C. WT cells behaved like the mutant parasites with Trolox (not shown). The values represent the mean ± SD of three independent experiments. For the –Trolox data sets, the p-values were calculated by paired two-tailed student's test. Statistically significant differences are marked (* p≤0.1; ** p≤0.05).
(TIF)

Figure S5 **Western blot analysis of transferrin-depleted medium.** Commercial holo-TF (100 ng) and 0.5 µl of the standard medium (corresponding to 100 ng TF) as well as of the fractions collected from the anti-TF column were subjected to Western blot analysis using the polyclonal antibodies against bovine TF. Fraction 1 was the FCS-free medium used for equilibration (not shown), fractions 2 to 4 represented TF-free medium whereas in fractions 5 and 6, TF was again detectable.
(TIF)

Table S1 **Primers used for the PCR analysis of** ***px I–II^−/−^*** **procyclic cells.**
(XLSX)
