# Supplementary figures and images for: Cytosolic Peroxidases Protect the Lysosome of Bloodstream African Trypanosomes from Iron-Mediated Membrane Damage
Source: PLoS Pathog. 2014 Apr 10;10(4):e1004075. doi: 10.1371/journal.ppat.1004075 (PMC3983053; doi:10.1371/journal.ppat.1004075)

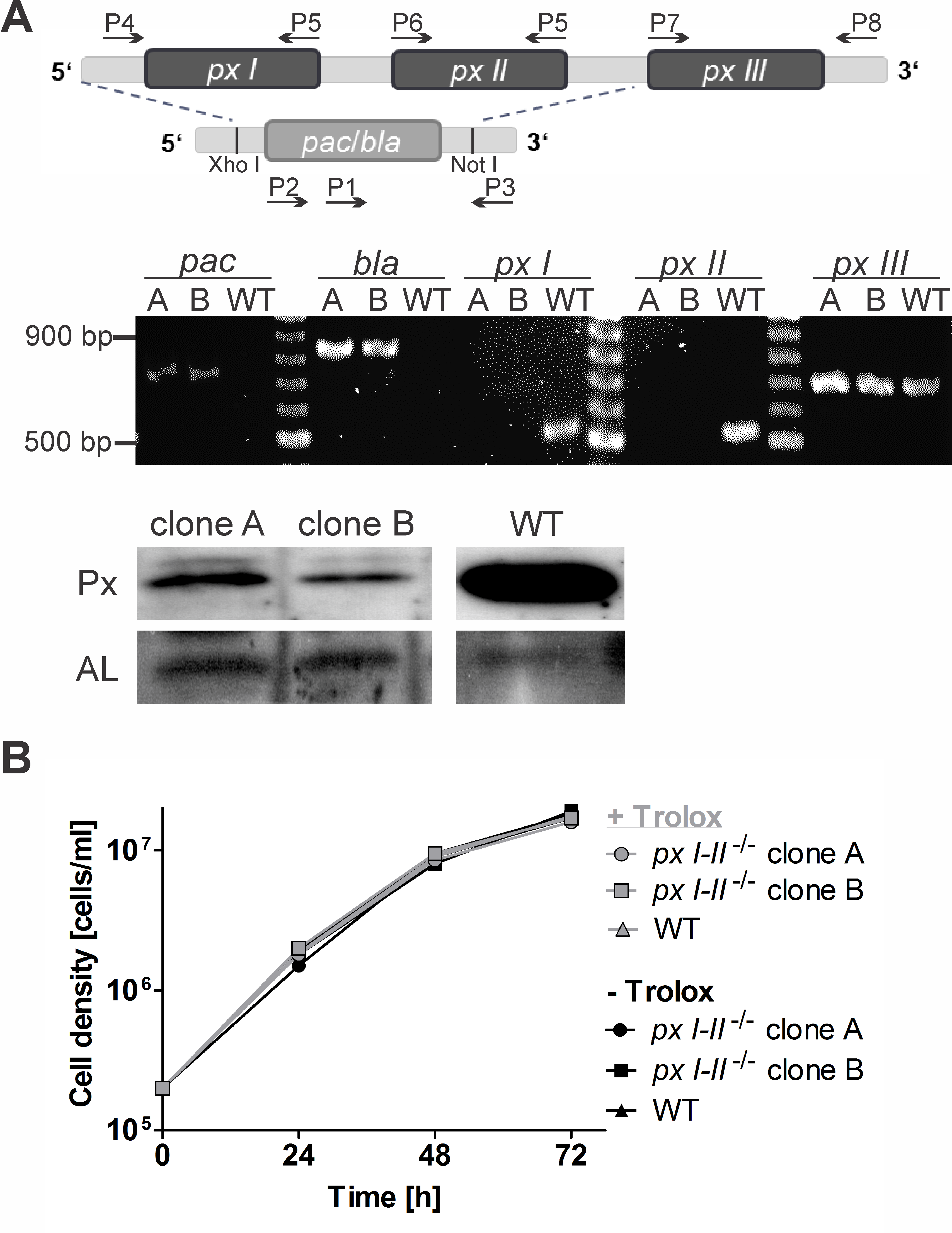

Supplement: Figure S1 — Generation and phenotypic analysis of procyclic px I–II−/− T. brucei . A. Upper part. Genomic px locus. The px I–II genes were replaced by transfecting the parasites with constructs containing a puromycin and blasticidin resistance gene, respectively, flanked by 5′ XhoI/3′ HindIII and 5′ PstI/3′ NotI restriction sites 5′ and 3′ of the resistance gene, respectively, generated previously [19]. Middle part. To verify the specific removal of the px I–II alleles and correct insertion of the resistance genes after the two consecutive transfections, genomic DNA of two px I–II−/− cell lines (clones A and B) and WT parasites was subjected to PCR with different primer pairs (pac: P1 and P3, bla: P2 and P3, px I: P4 and P5, px II: P6 and P5, and px III: P7 and P8). Lower part. Western blot analysis of the two px I–II−/− clones and WT cells against Px and aldolase (AL) as loading control. The remaining comparably weak band corresponds to the mitochondrial Px III. B. Proliferation of the px I–II −/− cell lines (clone A and B) and WT cells in the presence (+) and absence (–) of 100 µM Trolox. The data are representative of three independent experiments giving identical results. (TIF) [file ppat.1004075.s001.tif]

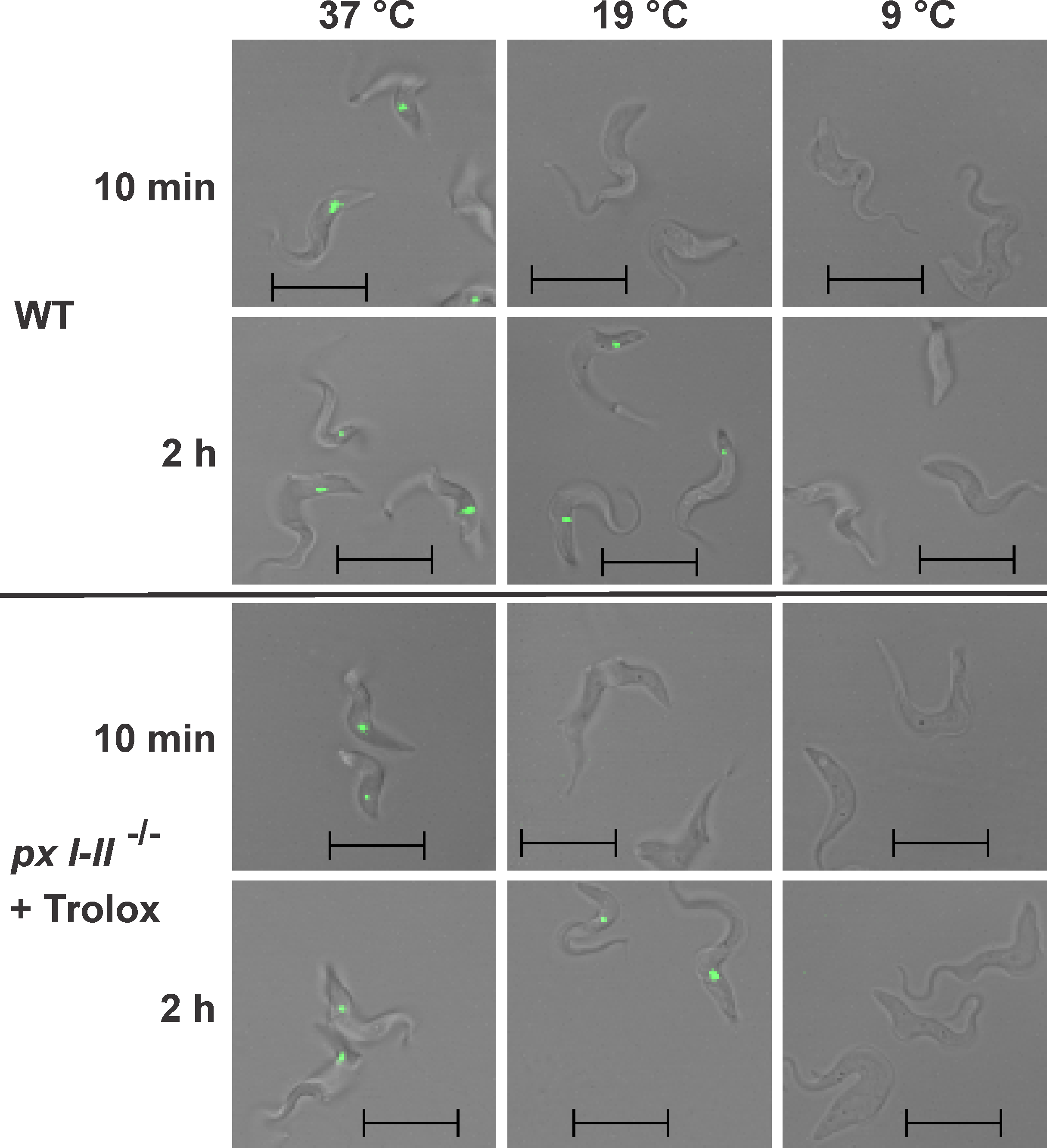

Supplement: Figure S2 — Temperature-dependent uptake of fluorescent dextran by WT and px I–II−/− BS parasites. Living cells were incubated in standard medium with 2.5 mg/ml Alexa Fluor-488 conjugated dextran at 37°C, 19°C, and 9°C for 10 min and 2 h. In the case of the px I–II−/− cells, the medium was supplemented with 100 µM Trolox. The major phenotype of the respective cell populations is displayed. At 37°C, already after 10 min, the whole cell population displayed lysosomal staining. At 19°C, after 10 min practically none of the cells showed a fluorescent signal, but after 2 h, the picture was indistinguishable from that at 37°C. At 9°C, no significant staining was observed. No difference was noticed between WT and mutant parasites. (Scale bar: 10 µm). (TIF) [file ppat.1004075.s002.tif]

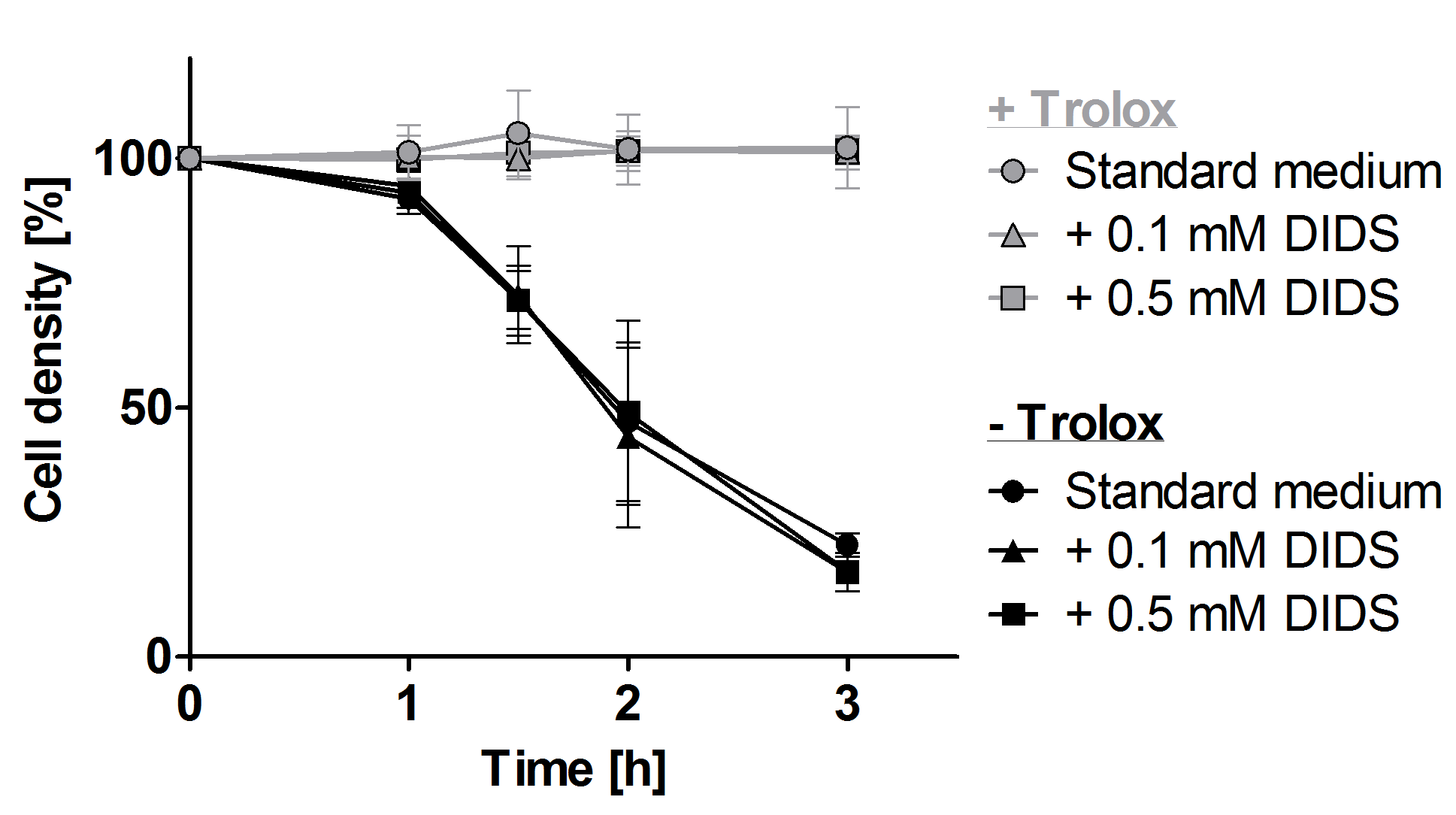

Supplement: Figure S3 — DIDS does not protect the px I–II −/− BS cells from lysis. The mutant cells were incubated in standard medium ±100 µM Trolox containing none, 0.1, and 0.5 mM DIDS. The data represent the mean ± SD of three independent experiments. (TIF) [file ppat.1004075.s003.tif]

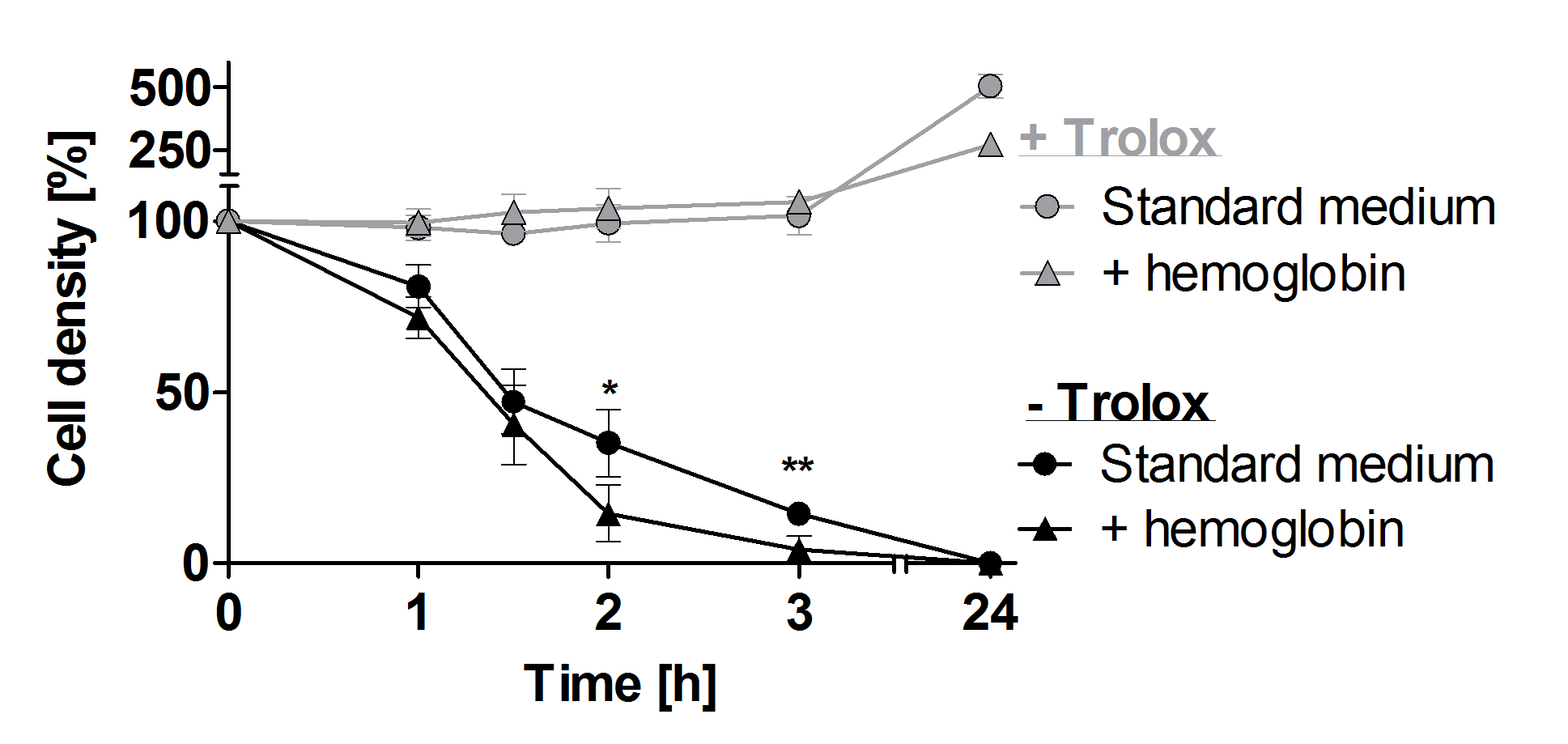

Supplement: Figure S4 — Supplementing the medium with hemoglobin slightly induces lysis of the px I–II −/− BS parasites. Cells were cultured in standard medium in the presence and absence of 100 µM Trolox and 16 µM hemoglobin at 21°C and subsequently incubated overnight at 37°C. WT cells behaved like the mutant parasites with Trolox (not shown). The values represent the mean ± SD of three independent experiments. For the –Trolox data sets, the p-values were calculated by paired two-tailed student's test. Statistically significant differences are marked (* p≤0.1; ** p≤0.05). (TIF) [file ppat.1004075.s004.tif]

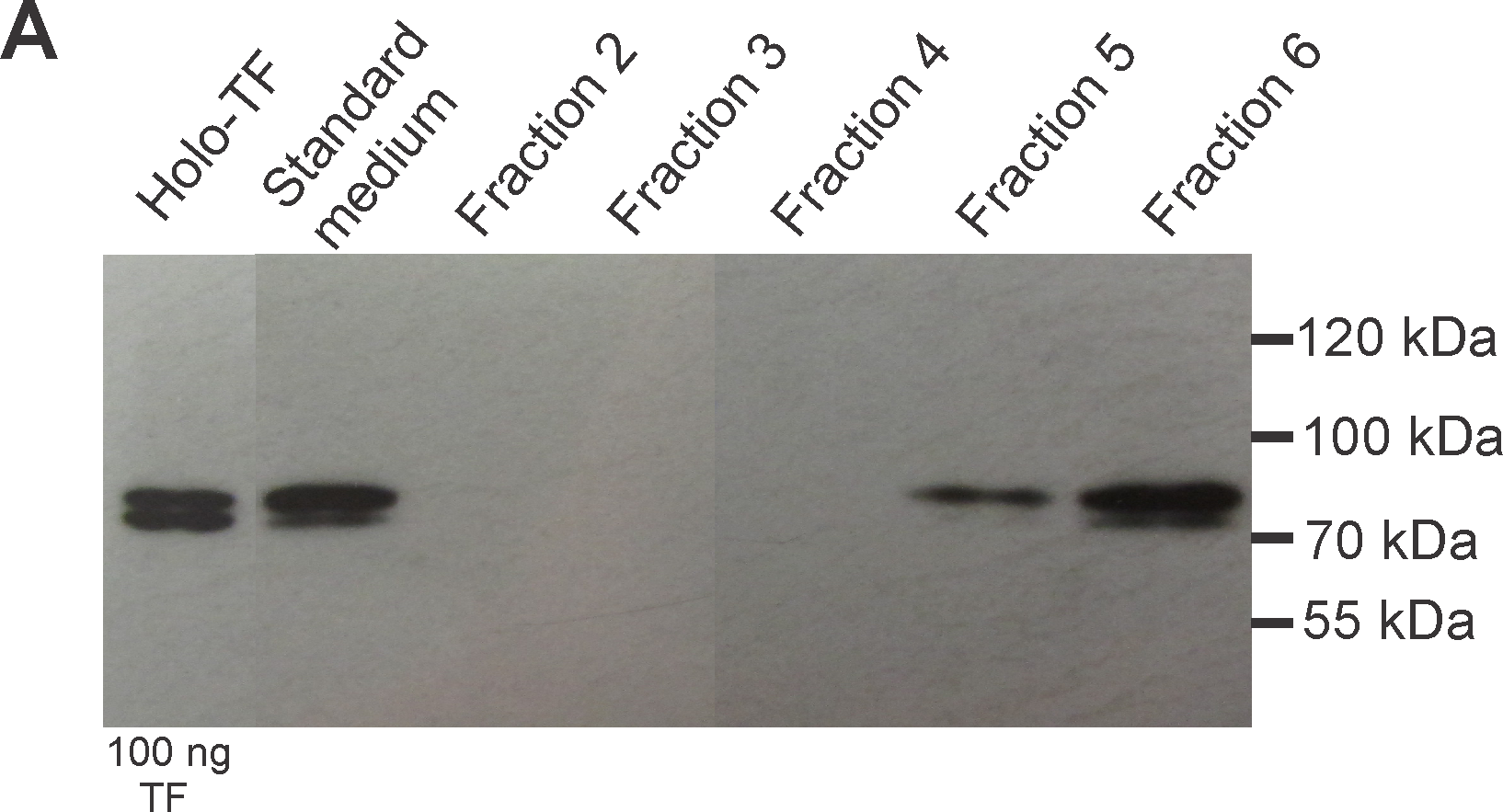

Supplement: Figure S5 — Western blot analysis of transferrin-depleted medium. Commercial holo-TF (100 ng) and 0.5 µl of the standard medium (corresponding to 100 ng TF) as well as of the fractions collected from the anti-TF column were subjected to Western blot analysis using the polyclonal antibodies against bovine TF. Fraction 1 was the FCS-free medium used for equilibration (not shown), fractions 2 to 4 represented TF-free medium whereas in fractions 5 and 6, TF was again detectable. (TIF) [file ppat.1004075.s005.tif]
